# Post-crisis Zimbabwe’s innovative financing mechanisms in the social sectors: a practical approach to implementing the new deal for engagement in fragile states

**DOI:** 10.1186/s12914-014-0035-6

**Published:** 2014-12-14

**Authors:** Peter Salama, Wei Ha, Joel Negin, Samson Muradzikwa

**Affiliations:** UNICEF Ethiopia Country Office, Africa Hall, PO Box 1169, Addis Ababa, Ethiopia; UNICEF Tanzania Country Office, Dar es Salaam, Tanzania; Sydney School of Public Health, University of Sydney, Sydney, Australia; UNICEF Zimbabwe Country Office, Harare, Zimbabwe

## Abstract

**Background:**

Donor engagement in transitional settings, complex emergencies and fragile states is increasing. Neither short-term humanitarian aid nor traditional development financing are well adapted for such environments. Multi-donor trust funds, in their current form, can be unwieldy and subject to long delays in initiation and work best when national governments are already strong. We reviewed the aid modalities used in Zimbabwe through the period of crisis, 2008–2012 and their results and implications. Literature review and case experience was utilised.

**Discussion:**

By focusing on working with line ministries in non-contested sectors to determine local priorities rather than following global prescriptions, pooling funds to achieve scale rather than delivering through fragmented projects, and building on national systems and capacities rather than setting up parallel mechanisms, the Transition Fund Model employed in Zimbabwe by UNICEF and partners in partnership with the Inclusive Government was able to achieve important results in health, education, social support and water services in a challenging setting. In addition, forums for collaboration were developed that provided a platform for further action. The initial emphasis on service delivery diffused much of the political delicateness that impeded progress in other sectors. The Zimbabwean experience may provide a model of innovative financing for countries facing similar circumstances.

**Summary:**

Such models may represent a new practical application of the Paris Principles, consistent with the major tenets of the 2011 New Deal for Engagement in Fragile States agreed in Busan. As we approach the Millennium Development Goal deadline, an over-arching, mutli-sectoral and independent evaluation of this approach is recommended in order to validate findings and assess broader replicability of this approach.

## Background

As the international community begins to focus on the next set of broad-based international development goals to follow the Millennium Development Goals (MDGs), increasing attention is being given to human security and to fragile states. The number of fragile states globally is in the range of 30 to 40. Taken together, such states account for: more than 20% of the world’s population (including a third of the poorest, living on less than a dollar a day); around half of the children dying before their fifth birthday; one third of those dying due to AIDS; and around 30% of maternal deaths [1]. Indeed, one of the key lessons learned from the MDG period has been that it will be difficult to achieve most of the MDGs without accelerated progress in fragile states [2].

In this paper, we aim to contribute to the evidence base on aid and development effectiveness in complex and fragile settings by using the case study of Zimbabwe, describing the types of aid modalities used in recent years, providing preliminary information on the major model used from internal and external reviews, and drawing initial lessons that may be applicable to other fragile states or complex emergencies.

There are many definitions and typologies of fragile states. A United States Agency for International Development paper, for example, classified fragile states as those which experience post-conflict, early recovery, arrested development, or deteriorating governance [3]. A definition proposed by the United Kingdom’s Department for International Development (DFID) describes fragile states as states that lack either the capacity, or the will (or both), to deliver core state functions for the majority of their people, including the poor [4]. One prominent typology is the Country Policy and Institutional Assessment constructed by the World Bank; countries are rated in the areas of economic management, structural reforms, social inclusion and public sector management. Countries in the bottom two quintiles on this multi-dimensional index are considered to be fragile states [5]. Zimbabwe consistently ranks at the bottom of these tables and classifies as a fragile state according to both DFID and World Bank definitions [6].

Until recently, discussions around best practice in aid and development effectiveness have tended to focus on stable development settings. The Rome Declaration and its successors, the Paris Declaration and the Accra Agenda for Action, all emphasized the need for country ownership, harmonisation of donor inputs, and alignment against national priorities as determined by recipient governments. The common modalities to implement such approaches were often instruments such as direct budget support, sector budget support, and Sector-Wide Approaches (SWAps). Bilateral donors and the International Financial Institutions often played a central technical and fiscal role in such situations including supporting the development of Poverty Reduction Strategy papers and medium-term expenditure frameworks. The United Nations system and civil society organisations tended to focus more on advocacy, capacity building, and providing support to policy reform. Increasingly, to assist governments in their planning and coordination in these settings, donors have endeavoured to provide more predictable, multi-year support and non-earmarked funds.

By contrast, in fragile states, the default approach has tended to be to adopt the humanitarian architecture and instruments. Such instruments – including the humanitarian cluster system bringing together UN agencies and international NGOs - have proven to be effective in delivering life-saving interventions in acute humanitarian crises. However, the duration of complex emergencies or state fragility may span many years and transformation of basic governance may take decades. The alternative development mechanisms, such as budget support and SWAps, have generally been considered inappropriate in such settings because fragile states lack the capacity and governance standards that generally facilitate such modalities being adopted [7]. In fragile states, national development frameworks or even sector plans are often absent. The commonly used model by development partners operating in fragile states can therefore be characterised as: limited funding over short time periods; project rather than budget support; use of parallel systems such as funding through NGOs and multilateral agencies, such as the United Nations and World Bank (including the use of multi-donor trust funds), rather than use of state implementers; and emphasis on humanitarian over development aid [5,8,9].

Recent research shows that this approach has serious limitations [9,10]. First, aid to fragile states is often too little too late. Donors have had a tendency to only begin delivering substantial aid to fragile states once there is a crisis. Second, volatile aid flows reduce rather than build recipient governments’ ability to implement projects and manage citizens’ expectations about public service delivery which could exacerbate instability and weaken the legitimacy of new governments at the exact moment when a new ‘social contract’ is urgently needed [11]. Third, obsessed with risk aversion, there is limited consideration by development partners of the costs of non-intervention. Donors have also tended to fund and support NGOs at the exclusion of government and then turned their attention to government strengthening late in the process [12]. Furthermore, the near exclusive emphasis on funding and support for NGOs, particularly in politically volatile contexts, can exacerbate tensions between government and NGOs which may be perceived by suspicious governments as partisan in their work within communities. Ultimately, the fragmentation that arises from many disparate projects is particularly difficult for weak governments to coordinate and manage.

Furthermore, commentators state that the global donor community cannot just assume that aid effectiveness in fragile states can be improved by simply moving from humanitarian modalities to those that are considered development mechanisms [13,14]. Early recovery situations are unique and require specific solutions based on flexibility and creativity. Rather than limited engagement until a state starts performing well in terms of policy and institutional capacity, recent findings tend to recommend more and earlier engagement with the recipient government as far as possible [9]. Some have characterised this as “shadow alignment,” [15] while others have called this “principled engagement” [16].

In 2007, taking into account these concerns, Organisation for Economic Co-operation and Development (OECD) countries adapted the principles for good international engagement in fragile states [17,18]. More recently, at the Fourth High-Level Forum on Aid Effectiveness in Busan in 2011, a number of post-conflict and fragile states as well as international organisations endorsed an agreement – the New Deal for Engagement in Fragile States – that gives clarity on how global actors can best engage in fragile states [1]. The New Deal emphasises building mutual trust, transparency, risk-sharing, use of country systems, strengthening national capacity, and timely and predictable aid. While the New Deal has highlighted the particular constraints faced by fragile states, the practical mechanisms for implementation remain unclear. Indeed, there is very little empirical evidence assessing performance of various aid modalities in fragile states; Waldman has noted that “there are far are more questions than answers and far more theory than experience” [3].

There is, therefore, an urgent need to learn more about development and aid effectiveness in fragile states and to move beyond agreeing on theoretical principles to documenting recent country experience. In addition to providing a test of the Busan commitments, such information may be timely as, recently, a number of development partners have reiterated their intention to invest substantially in fragile states. Already around 30% of all Official Development Assistance is spent in fragile and conflict-affected countries. The analysis presented is based on a review of the published and the grey literature on programme models in fragile states, using the Google Scholar search engine with key words, ‘fragile states and financing instruments,’ a review of the major donor mechanisms used in Zimbabwe during the period 2007–2012 with a particular focus on the transitional funding mechanisms, and on the direct personal experience of the authors in Zimbabwe and more than 20 other complex emergencies and/or fragile states. One of the authors played a direct role in the conceptualization, design, and implementation of the transition fund model. Two of the other authors are involved in monitoring and evaluation of this model and other programmes in Zimbabwe and, one author, although knowing the Zimbabwean context, is an academic with no direct role in implementing programmes in Zimbabwe. No ethical approval was needed for this study which is presented for debate.

### The Zimbabwean context

Following independence in 1980, Zimbabwe achieved major improvements in coverage of basic social services and also a sharp decline in disparities. Due to increasing political instability around the turn of the century, however, the macroeconomic environment collapsed with a 48% decline in gross domestic product between 2000 and 2008, hyperinflation peaking at 231 million % in 2008, and the unemployment rate reaching 80%. Meanwhile, HIV prevalence rates reached close to 30%, further reducing productivity and heightening health care costs.

These factors led to the near total collapse of basic social services in 2008. Zimbabwe, once the food basket for the southern African region, became a net importer of food with more than 5 million people dependent on food aid. Maternal mortality rates more than doubled between 1990 and 2008 as only half of the pregnant women gave birth with the assistance of skilled health personnel. The education system was also in crisis and, in 2008, government schools effectively closed for a large part of the academic year as teacher salaries dropped to the equivalent of less than US$2 per month. Perhaps the most dramatic example of the deterioration in the social sectors, however, was the cholera outbreak of 2008 and 2009 which led to 98,531 cases and 4,282 deaths.

After a disputed election in Zimbabwe in 2008, the three political parties agreed to form the ‘Inclusive Government’ (also known as the ‘Government of National Unity’) under the Global Political Agreement signed in September 2008. Under the Global Political Agreement, the former ruling party - ZANU-PF - retained control of the Ministries of Defence, Foreign Affairs, Mines, Agriculture, Local Government and Justice, among others. The opposition Movement for Democratic Change – Tsvangirai (MDC-T) took control of Finance, Economic Planning and the majority of the social sectors (Health, Education, Water, Labour and Social Services) with the other opposition group, the Movement for Democratic Change –Mutambara (MDC-M), given smaller portfolios. The relative political stability that ensued under the Inclusive Government ushered in a period of fragile, but tangible, economic and social recovery.

**Figure 1:**
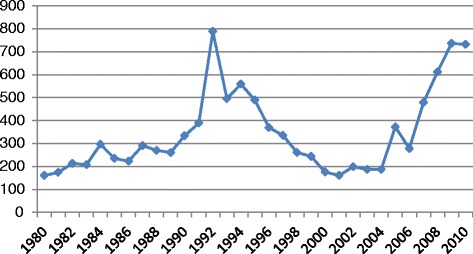
**Disbursed official development assistance from all donors to Zimbabwe in current US dollar millions, 1980 to 2010 [** 20 **].**

A national development framework, the Medium-Term Plan was endorsed by cabinet in 2011 and replaced the more emergency-oriented Short Term Emergency Response Plan. With the establishment of the power-sharing government and a consensus that the arrangement, although imperfect, represented the best chance yet for Zimbabwe to emerge from the political, economic and human crises of the previous decade, development partners sought to re-engage albeit cautiously. Despite the ‘considerable international scepticism’ about whether the political arrangement could succeed [19], since the swearing-in of the transitional government, donors have committed substantial amounts of aid returning it to a level last seen in the early 1990s (Figure 1). The bilateral donors which contributed the most in 2010, included the United States, United Kingdom, European Union and Australia [20].

Despite this progress, due to the targeted sanctions imposed during the last decade and consequent lack of official engagement between the Government of Zimbabwe and international donor community, very limited direct donor funding has been provided to the Government of Zimbabwe and, until recently, humanitarian funding streams had been the dominant source of support. In addition, the International Financial Institutions have not fully re-engaged in Zimbabwe due to the political situation and the large amount of arrears (according to the World Bank country profile this amounted to 70% of gross domestic product at the end of 2012) owed by GOZ to these institutions. Overall, the aid coordination mechanisms have also remained contentious and relatively non-functional with multiple parties and line ministries vying for control and continued reluctance of some OECD governments to engage directly with government.

### Description of financing approaches

The choice of financing mechanism used in fragile situations is critical as it affects how needs are defined and prioritized, donor behaviours, accountability and capacity development approaches [21]. However, no consensus exists as to which form of lending or granting instrument is best for fragile settings in order to achieve multi-faceted development goals. One of the most popular mechanisms for delivering aid in such contexts is the multi-donor trust fund (MDTF). However, multi-donor trust funds historically have a mixed record [22], are often designed and created outside the country or context in which they will be implemented, suffer from a lack of clarity on goals and operational structure, and work best when recipient governments are strongest – the characteristic least likely to be present in fragile states. In addition, a recent review concluded that there is a lack of rigorous and independent research on multi-donor trust funds [23]. Published literature on other financing models for fragile states is even more rare.

During the period 2007–2012, several models of financing were developed in Zimbabwe, all with the objective of increasing predictable financial flows to the country while the sanctions regime constrained the ability of partners to fund the government directly.

The characteristics of three main types of mechanisms used in Zimbabwe during this period are summarized in Table 1 [24]. We discuss the major advantages and disadvantages below:

1. Multi-Donor Trust Fund: These funds are generally managed by the Development Banks with Ministry of Finance as the government counterpart. A policy committee acts as the steering group with funds administered according to Bank procedures and implemented through tendered sub-contracts to NGOs and private contractors. An example of this mechanism was the Zim-Fund for Water and Sanitation managed by the African Development Bank. These funds follow a tightly defined scope of work and the administrative rules of the IFIs that manage them. The strengths of the funds were that: they had the capacity to mobilize pooled funding from a range of donors; could draw upon the technical resources of the development banks involved; involved transparent tendering and procurement mechanisms; attracted private sector and civil society partners; and had the potential to reduce transition costs. The challenges in the Zimbabwe context where government was highly divided and polarized were that: the counterpart ministry was the Ministry of Finance rather than relevant sectoral ministries resulting in coordination challenges with sectors in which the funds were developing projects; significant delays in procurement due to stringent processes; limited use of government systems; and a tension between a focus on ‘quick wins’ and building sector capacities.
2. Global Funds, Basket Funds, and Pass-Throughs: These funds have generally been managed by the United Nations Development Programme in Zimbabwe with a coordination committee determining priorities and the United Nations acting as a pass-through. Funds are administered according to United Nations procedures and implemented through several United Nations agencies and civil society organisations. An example of this mechanism is the Global Fund portfolio on HIV, tuberculosis and malaria. Here the United Nations Development Programme acts as an administrative agent with limited technical role. The strengths of these funds are: the potential to harmonize various actors; a reduction in overhead costs; and involvement of a full range of stakeholders. The challenges are that: global and not local priorities tend to determine the size of funding streams; competition by various constituencies for resources may actually increase transaction costs; the limited use of government systems with procedures determined at global level; and coordination, technical support and fiduciary risk management accountabilities are unclear (since the United Nations only administers the funds) and, with many sub-grantees, these risks are significant.
3. Transition Fund Model: is described in detail below.

**Table 1:** Basic typology of different pooled funding mechanisms and defining characteristics*

	**Pooled funds from contributing donors agreed and defined in Zimbabwe**				**Global pooled fund or thematic**
	**Multi-donor trust fund (Bank)**	**Transition fund**	**Common fund (Pass through)**	**Simple basket**	**Request or allocation from global pooled fund**
**Alignment to National Priorities**	Aligned to national Government priorities (not necessarily sector policies or strategies)	Aligned to national Government strategies and multi-year plans. Government contribution	Aligned to national policies and strategies and priorities	Aligned to national priority	Aligned to national priorities (with some conditions set by the global fund requirements)
**Coverage**	Have national coverage relevance e.g. studies	Implemented on national scale through national structures	No national coverage (but could be expanded)	National coverage and relevance	Not necessarily
**Operational Framework**	Arrangements include standard legal agreements with all donors, which specify governance procedures covering trust fund management, operational and financial reporting,	Policies and procedures agreed, e.g. Joint Statement of Intent, Code of Conduct, administrative procedures meets agency requirements	MOU signed between agencies, administrative arrangements meet Un agencies’ requirements	Project document signed between agency and Government, meets all agency requirements	Request submitted and/or allocation made from global fund
					Implemented through single or multiple UN, Government, or NGOs guided by agreements
**Accountability**	World Bank has financial management, technical oversight and accountability for ensuring high quality results	Agency is fund manager and has technical oversight management and implementation responsibilities for results	UN agency identified to manage fund on behalf of other agencies with only financial accountability (meets agency requirements)	One UN agency as fund administrator, technical oversight and implementing agency	Agency identified in country to manage fund and co-ordinate it through multi-stakeholder forum
			Multiple implementing UN agencies and NGOs according to defined roles and responsibilities		
**Un-earmarked**	Bank-administered MDTFs do not allow donors to earmark funds	Generally Unearmarked	Generally unearmarked	Unearmarked	Unearmarked
**Management and Administration Costs**	7% overhead (2% + 5%)	12% overhead (5% + 7%)	1–3% overhead for fund administrator, 7% for implementing UN agencies	12% overhead (5% + 7%)	3% (ERF) or 7%–12% overhead
**M and E**	M and E developed through	Monitoring and evaluation linked to national targets, research component, independent review	Developed for individual agencies or programs	Developed against workplan and results of project	M and E framework developed to guide all implementing partners and/or indicators developed by project using template/guidelines
**Examples**	A-MDTF	HTF	Expanded Support Programme (ESP)	COPAC	CERF
	P-MDTF (Zimfund)	ETF-II			H4 + *
					(ERF)
		CPF	Emergency Response Fund (ERF)		Global Fund

*With permission [24].

### The transition fund model

#### Genesis

From 2007 to 2010, UNICEF managed a multi-donor pooled fund, known as the Programme of Support, to provide assistance to the 1.5 million orphans and vulnerable children in Zimbabwe. Prior to the programme’s launch in 2007, just as in many HIV hyper-endemic countries with an orphan crisis, a major scale-up of predominately NGO-implemented and donor- driven projects had occurred. In Zimbabwe, each major international donor had been supporting a group of NGOs to deliver services on a project basis to orphans and vulnerable children. Prior to 2007, the government ministry mandated to support social protection, the Ministry of Labour and Social Services (MOLSS), had had a very limited role in this system and very little capacity to provide stewardship over the complex set of programmes and actors. In particular, there was no overall social protection policy or framework, no standard package of services, and little monitoring and quality control. One of the objectives of the Programme of Support was to support the MOLSS in its policy, standard setting and monitoring role as well as to contribute to donor harmonisation and alignment.

In early 2010, the Programme of Support was formally evaluated using the OECD criteria and scored a B rating. The evaluation noted that more than 400,000 children had been supported with services and that the flexible approach adopted had allowed the support to continue even through a major national humanitarian crisis. However, the evaluation also noted areas for improvement including on quality of services delivered and the need to document and utilize the lessons learned. The evaluation team concluded that the next iteration of the programme would have a key role to play in the transition towards a more comprehensive national social protection framework and programme.

Taking lessons learned from the programme evaluation, a new aid mechanism more closely adapted to the complex environment was developed for social protection and the other major social sectors. Known as the Transition Fund, the objective of the mechanism was to address the central development challenge for Zimbabwe at that time: the need to find a mechanism that brought partners together in a programme rather than project approach, in order to obtain national scale impact under line ministry leadership, while also addressing the major donor and political restrictions on the channelling of funds directly through government.

The model contained the following features:

- Agreement between line ministry, donors and the secretariat on the major objectives and national targets of the programme which were fully aligned with broader sectoral objectives and targets.
- Setting up of a steering committee comprising relevant government ministries, bilateral donors, and United Nations and civil society partners that was co-chaired by a line ministry and a donor representative, with the secretariat managed by UNICEF.
- Policies and priorities of the line ministry providing the basis for the scope of work.
- UNICEF managing the programme and all financial resources on behalf of the specific line ministry, through pooled or ‘aligned’ funds*^a^, in accordance with its own rules and regulations.
- The scope of work generally including a major emphasis on service delivery at national scale with a focus first, on arresting the decline, then on accelerating progress against the relevant MDGs, as well as efforts in policy reform and capacity building.
- Partnering with other United Nations agencies, private sector and civil society organisations based on comparative advantage and value for money, and generally through competitive selection processes.
- Investing in robust monitoring and independent evaluation mechanisms to ensure high degree of accountability.

Based on the initial Programme of Support prototype and lessons learned, similar transition programmes were launched in other social sectors. The Educational Transitional Fund was launched in mid-2009. The Child Protection Fund was designed and launched in 2010. This fund included the first national scale social cash transfer programmes integrated with case management and justice for children services. In addition, smaller pooled funds have been launched for urban water, sanitation and hygiene and for the Essential Medicines programme. The latter has now been integrated into the Health Transition Fund launched in 2011. In total, more than $800 million of development assistance has been committed through these transitional funds by more than ten bilateral donors through 2015 (Table 2).

**Table 2:** **Overview of transition funds managed by UNICEF in Zimbabwe**

**Sector**	**Name**	**Duration**	**Scope**	**Funding levels (USD)**	**Major donors**
Child Protection	Programme of Support, Child Protection Fund	2007–2010 and 2011–2014 (phase 2)	OVC programmes evolving into integrated child and social protection including cash transfers, justice for children and case management	Phase 1: 70 million	UK
					Germany
				Phase 2: 80 million	Sweden
					NZ
					Netherlands
Urban water, sanitation and hygiene	Emergency rehabilitation and risk reduction	2009–2012	Urban WASH rehabilitation focused on small towns	40 million	Australia
					UK
					ECHO
Rural water, sanitation and hygiene	Water and sanitation transition fund	2011–2015	Rural WASH including borehole drilling, rehabilitation, new operations and maintenance models and community-led total sanitation	50 million	UK
Education	Education transition fund	2009 -2011 and 2011–2014 (phase 2)	Learning materials and textbooks evolving into focus on quality of and access to basic education including a new school grants programme, comprehensive curriculum review and support for second chance programmes for out of school youth	Phase 1: 50 million	EC
					Norway
				Phase 2: 70 million	Finland
					Netherlands
					UK
					Germany
					OSISA
					US
					NZ
					Australia
					Japan
					Sweden
Health and Nutrition	Health transition fund	2009–2011	Essential medicines evolving into comprehensive MNCH and health systems support, including essential medicines, human resources, and support to recurrent costs at health facility level	Phase 1: 70 million	EC
		2011–2015 (phase 2)			UK
				Phase 2: 400 million	Canada
					Sweden
					Ireland
					Australia
					Norway

**Figure 2:**
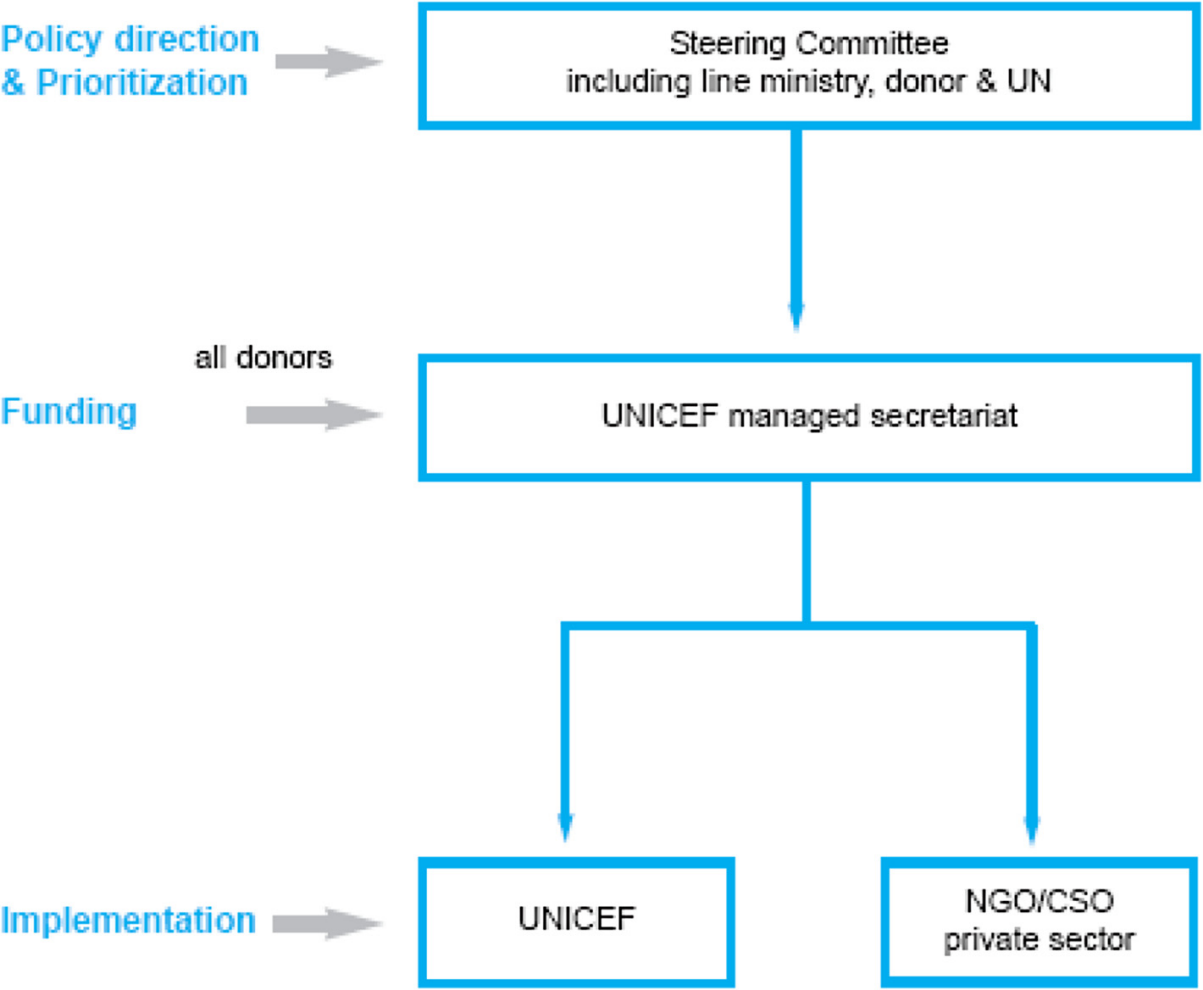
**Structure of the transition funds.**

Although following a similar basic structure (Figure 2), each fund operates in a slightly different manner depending on the specific sector dynamics. The steering committee, co-chaired by a senior government official in that sector (such as the Minister or Permanent Secretary) and a donor representative, helps to determine the priorities for the fund and ensures full alignment with the national sector plan. In fact, in all sectors in which they are operational, the transition funds are providing the major source of non-salary funding for the national programme in that sector as well as the major donor coordination platform. The steering committee, which generally meets monthly, consists of all major stakeholders in that sector, including representatives from all major donors (including those not contributing to the fund directly), other relevant ministries, international and national NGO partners and relevant United Nations agencies. Decisions are generally made by consensus. Given the donor restrictions, funds are managed by UNICEF and expenditure is made against the agreed plan and budget lines. Because funds are generally non-earmarked and pooled, the steering committee can re-prioritise if needs change or another donor, not providing funding through the pool, decides to cover an area originally covered by the fund. All United Nations financial and procurement procedures are followed, including competitive tendering for procurement of goods and services. Implementation is through sub-national government structures directly, where they are functioning, or through the United Nations, private sector or civil society partners. Selection of the implementing partners is generally made by a sub-committee of the steering committee consisting of government, United Nations and donor representatives. Progress reviews are commissioned annually and mid-term and end of cycle evaluations are conducted by independent entities. One of the critical differences in implementation of these funds has been the unique and multiple roles entrusted to UNICEF; UNICEF acts as the secretariat of the mechanism, manages funds and procurement, assists the relevant ministry in donor and programme coordination as well as provides additional technical capacity as required in specific areas. These multiple roles, which of course go well beyond the conventional role of fund manager, were the subject of much debate in the design of the funds, most notably on how to manage issues surrounding potential conflict of interest. UNICEF was chosen as the fund manager and secretariat because of its track record in the relevant sectors (health, education, wash, social protection), expertise on procurement, existing good relationships with both line ministries and donors and, as the largest and most well-staffed of the United Nations agencies in Zimbabwe, its ability to manage risks of such large scale operations in a difficult political context. Other partners particularly those in the private sector were considered for certain elements (procurement, payment of health workers) but none were deemed to be able to cover the full range of activities. In addition, the operational environment for NGOs was difficult. In sum, few other partners were deemed realistic alternatives to UNICEF.

## Discussion

**Figure 3:**
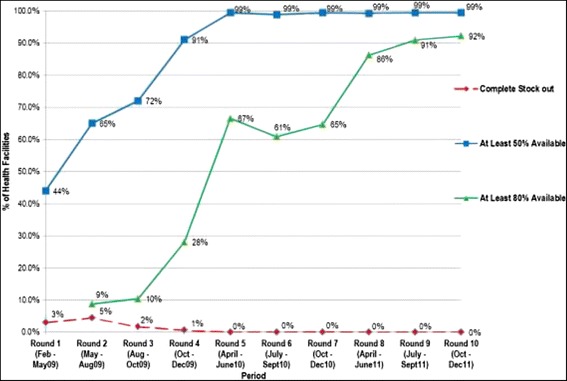
**Trends in availability of selected essential medicines in health facilities after implementation of the essential medicines programme in 2009.**

For the purposes of this paper, we are focusing on the results of the transition fund model- which was the major source of large-scale funding for the education, wash and social protection sectors through this period and for non-HIV health funding. In the first few years of implementation, the transitional fund mechanisms have achieved the following results:

- More than 24 million textbooks (as well as stationery, sporting kits and storage cabinets) have been provided to all 8,000 primary and secondary schools in all core subjects, taking the ratio of pupils to textbooks from 10:1 to 1:1.
- Around 500,000 orphans and vulnerable children were supported with multi-sectoral programmes that improved access to services, participation and enjoyment of basic rights.
- An additional 500,000 orphans and vulnerable children have had their school fees paid annually.
- The first national social cash transfer programme for the extremely poor has been initiated, reaching 20,000 households.
- All 1,400 primary health care facilities have been consistently supplied with the basic package of essential medicines and all vaccine requirements dramatically reducing stock-outs (Figure 3).
- All 20 major urban centres were supplied with water treatment chemicals and technical support for rehabilitation of water treatment plants to bring the cholera outbreak under control.

### Results in the health sector

In terms of detailed results in the health sector, the recent Multiple Indicator Cluster Survey carried out by the Zimbabwe National Statistics Agency shows positive recent trends in both the reduction of under 5 and maternal mortality [25]. The authors of the report attribute these changes to the programmes supported through the Health Transition Fund. The main justification for their conclusion is that no other major new initiatives have been implemented in the sector that could explain this progress. In addition, process indicators improved in the period between the two nationally representative Multiple Indicator Cluster Surveys of 2009 and 2014 and much of this progress occurred in recent years. These improvements included: expanded programme on immunisation coverage (37% to 69%); exclusive breastfeeding (26% to 41%); post-newborn care coverage (12 to 85%); skilled birth attendant coverage (60% to 80%), four ante-natal sessions coverage (57% to 70%) and postnatal care of the mother (27% to 78%). Furthermore, there were documented declines in disease specific morbidity for malaria, HIV and vaccine-preventable diseases and that there have been no major outbreaks of cholera or measles since the Health Transition Fund commenced. All evidence points to the fact that the Health Transition Fund has made a major contribution to these results.

The findings of the Mid-Term Review of the Health Transition Fund in July 2014 conducted by Liverpool School of Tropical Medicine also provide strong evidence for system wide improvements, albeit modest in some areas. In terms of human resources for health, vacancy rates amongst health workers have declined by 5% (from 21% to 16% during the period 2012–2014) and, since 2012, 2000 new midwives have been trained and deployed. The number of doctors at district level has doubled and the total number of village health workers has increased by 4000. In terms of reductions of inequality, user fees had been a major financial barrier to access for the most vulnerable population. One of the pillars of the Health Transition Fund, the health service fund has been providing primary health care clinics and district and provincial hospitals monthly stipends for their running costs. As a result 94% of PHC clinics and 82% of hospitals are now providing free care to pregnant women and children under 5. Finally, in terms of government allocations to the health sector during this period- budgets allocated doubled from US$174 million in 2010 to US$381 million in 2013 but disbursement varied from 62% to 86% of allocated amounts. However, at 9.9% of the total national budget, government allocations to the health sector are still well below the 15% target set by the Abuja declaration.

It is important to note that in preceding years, the donor approach had emphasized two alternative models- relatively small scale individual humanitarian projects delivered by NGOs and more vertical mechanisms, the largest being the Global Fund in the health sector. By taking the health sector as an example, it is possible to compare and contrast the potential results that may have been achieved using one of these alternative models, assessing them on the criteria of scale of results, adherence to national priorities, building on national capacities and systems, and risk management.

First, we believe that such results at national scale would have been very difficult to achieve using other mechanisms. Because of fragmentation, lack of economies of scale, or a vertical focus, neither model had managed to prevent further deterioration in the health sector (or any of the other social sectors), which had collapsed in 2008. By contrast, the Health Transition Fund managed to pool resources together, for the first time on that scale, against national sectoral targets, thus contributing to stabilizing the sector and, according to recent outcome and impact level data, may have contributed to significant progress on the health-related MDGs.

Second in terms of following national priorities, in this politically-charged environment of sanctions, the donors had previously favoured humanitarian mechanisms precisely because such mechanisms allowed them to implement projects without having to formally consult government. Global Funds had involved government officials in national mechanisms such as the Country Coordinating Mechanism but within the strict confines of Global Fund priorities at the time-determined in Geneva-particularly HIV and to a lesser extent tuberculosis and malaria. Furthermore as a disease-specific funding stream, the Global Fund did not have the latitude, flexibility or resources, to truly address broader health system issues such as human resources shortfalls and the stock-outs of critical essential medicines which had resulted in collapse of the sector and were the over-riding national priorities at the time. By contrast the transition funds were designed by teams comprising members of in-country partners including senior government officials from line ministries, donors and the United Nations. These teams were already involved in the relevant sectors and fully cognizant of sectoral priorities.

Similarly in terms of building on national capacities and systems, neither alternative model was fit for purpose in this regard; the humanitarian actors because of their perceived need to remain independent of the Government of Zimbabwe as well as the short-term nature of their funding and the global funds because issues such as capacity building were seen as beyond their remit, vested interests within the Country Coordinating Mechanism maintained a narrow focus on HIV, and working through national systems was perceived as somewhat risky. By contrast the health transition fund was designed in such a way that systems building was a core focus of 3 of the 4 pillars and the pooling of resources allowed for flexibility in approach. In addition, a code of conduct signed by all Health Transition Fund steering committee members committed them to supporting national priorities and all of the over-arching goals of the fund, not merely those of interest to any one donor agency or implementing partner.

Finally, in terms of risk management, because the locus of decision making for both alternative mechanisms (donor country capitals or executive board of the Global Fund) remained predominately the donor capitals it was more difficult for local actors to adapt to the rapidly changing political context in Zimbabwe and to assess and re-assess risk. By contrast, once resources had been committed to the Health Transition Fund, the steering committee provided a forum for analysing risk and finding local solutions to addressing risks. This was facilitated by the fact that funding was pooled and hence any individual donor felt that their risks were also shared. In addition, the large, combined field presence of the major health sector partners on the steering committee and commitment to regular monitoring and independent evaluation reassured donors that problems occurring would be identified quickly. This in turn contributed to less risk aversion and support for programmes such as large scale health retention schemes which were previously considered too risky by most donors.

### Collateral benefits of transition funds

In addition, in terms of added value, other ‘collateral’ benefits of this model are also becoming evident. First, prior to the existence of the steering committees of these funds, there were no fora for systematic sectoral-level engagement between donors and the relevant line ministries. The steering committees have thus provided a strong platform for sectoral-level coordination, harmonisation of donor positions and, notwithstanding the sanctions environment and associated restrictions, alignment behind government policies, programmes and priorities. In every case where a transition fund exists, the fund has become the dominant coordination mechanism for major donors to that sector. Such fora have assisted in building relationships between donors and line ministry officials which in some cases had been non-existent or problematic for several years.

Second, such mechanisms have provided a more structured approach to supporting longer term priorities in each sector, reducing fragmentation in the process. The transitional funds have assisted in the evolution from a purely humanitarian focus to ‘humanitarian plus,’^b^ and now to recovery and transition. Increasingly, and partly as a direct result of these mechanisms, donors are now providing longer term and more predictable funding for Zimbabwe. Third, through efficient management practices, economies of scale, and transparent procurement processes, the funds have been able to achieve impressive value for money. For example, in the areas of procurement of water treatment chemicals and medicines, taking advantage of global and regional UNICEF contracts, major savings have been made. Under the Educational Transitional Fund, the procurement of textbooks alone saved more than US$50 million allowing the purchase of the national requirement of both secondary and primary books to remain well within the original budget envelope for primary books alone.

Fourth, in each of these sectors, the transition funds have created a powerful platform to advocate for critical sectoral policy reforms. In education, agreement has been reached to embark on a comprehensive national review of the curriculum as well as also providing the forum for the development of a Medium-Term Education Sector Plan, now approved by Cabinet. In health, one of the main objectives of the Health Transition Fund is to facilitate the removal of all user fees for pregnant women and children under 5 by the end of 2013. In addition, large scale support to supplement the salaries of government health workers has resulted in the return of doctors to many district and rural hospitals. The Child Protection Fund has resulted in the first national scale social cash transfer programme and is accelerating the national momentum around developing a comprehensive social protection framework. In water and sanitation, the reform of national inter-ministerial Water Sanitation Hygiene coordination structures has been implemented and the new national water and sanitation policy is being drafted. The transition funds have also aligned with the objectives, implementation and targets of the country’s Medium Term Plan and provided an effective coordinating platform for the monitoring and evaluation of Medium Term Plan targets in the social sectors – the same targets that are reflected in the transition funds.

Finally, by raising the profile of the social sectors themselves, the transition funds have contributed to the national debate around the need to increase the government budget commitments to these sectors as well as to the momentum for taking full national ownership over these programmes as soon as is feasible. For example, when a local newspaper recently falsely reported that UNICEF was withdrawing its financial support from the Basic Education Assistance Module (the social protection programme that provides school fees for more than 500,000 orphans and vulnerable children annually), a major national debate ensued on the need to rely on national resources to fund such critical programmes. We have witnessed similar debates in relation to national contribution to antiretroviral medication requirements and to supporting procurement of the water treatment chemicals that ensure safe water in urban centres. Such debates have resulted in formal parliamentary reviews ultimately resulting in increasing treasury commitments to the social sectors from 33 per cent of the total budget in 2010 to 39 per cent in 2012 [26].

All of the transition funds have been subjected to annual review, conducted by an independent entity following terms of reference defined by the steering committee of the fund. The Educational Transitional Fund has benefited from two such reviews in its first phase of operations. Both reviews found the Educational Transitional Fund to be a relevant project that was well managed, effective in meeting its objectives, and cost effective. In particular, it was noted that the textbook procurement, which resulted in a cost saving through transparent procurement processes, effective negotiation and economies of scale, ‘had delivered far in excess of initial expectations…’ Similar findings have been noted for the essential medicines programme and for the Child Protection Fund as well as the urban water sanitation hygiene programme.

In terms of the essential medicines programme, in its 2011 assessment of DFID’s support to the health sector, the United Kingdom’s Independent Commission for Aid Impact noted positive results in all areas assessed (objectives, delivery, impact, and learning) [27]. The report commented that DFID helped to avoid the total collapse of the health system during the crisis years by providing essential medicines and supplementing the salaries of key staff. The essential medicines programme was also credited with obtaining economies of scale and value for money. Several evaluations of the urban water sanitation hygiene programme have been commissioned by AusAID – the major donor. All have found the programme to be relevant, effective and efficient and have credited it as a major factor in bringing cholera under control [28]. In relation to the Child Protection Fund, the latest donor review in 2012 found the combined cash transfer and child protection programme to be functioning well scoring an ‘A’ overall for meeting all expectations. The review emphasised the strong national ownership of the Child Protection Fund programme.

### Discussion and lessons learned

#### Importance of an understanding of the political economy and context

An understanding of the specific history of the social sectors in Zimbabwe and particular challenges facing the current senior government officials during this period was critical in order to tailor the support of the international community appropriately. From the period of Independence in 1980 to the mid-1990s, Zimbabwe had had very strong government-led social sectors with only a modest reliance on international donors, United Nations agencies or NGOs. And despite serious underinvestment in the social sectors for more than a decade prior to the crisis of 2008–9, large scale emigration of skilled workers, and a serious decline in capacity that occurred at all levels during this period, the senior civil servants in some line ministries retained a strong tradition of public sector leadership and of managing for results. However, because in the past significant external resources had not been required, the majority of ministers or senior civil servants were neither entirely familiar with the international aid architecture nor had they had much experience of coordinating multiple stakeholders and balancing various external interest groups. These challenges were also compounded by the fact that the MDC-T and MDC-M ministers, being members of the former opposition movement, had had virtually no experience of governing or running large sectoral ministries.

One of the major reasons for the relatively successful implementation of these mechanisms has been an in-depth understanding of the priorities, processes and players in each of the sectors. The transition funds were designed in-country, in a collaborative manner, by existing major stakeholders with expertise and experience in those sectors, with priorities based on existing sector plans. In addition, the mechanisms, although following a general prototype, have been tailored to the sectoral context and helped support national institutions and systems as well as sectoral line ministry leadership. Finally, in a complex political environment, cognizance of the historical context and sensitivity to perceptions in a highly polarised political environment has been critical [29]. Thus one of the major pitfalls of multi-donor trust funds, that of funds being born in a vacuum, has generally been avoided from the outset [23].

In addition, respect for the multi-lateral nature of the UN system and its ability to transcend, and *be perceived to transcend* party politics has been a critical factor in the successful implementation of the funds. The UN agencies have played an important brokering role between donors and the government, between NGOs and the government, and even between different government ministries led by opposing parties. The Independent Commission for Aid Impact report on the health sector commented that partners found ‘working with the UN in Zimbabwe at this time to be effective, given the political situation.’ A small group of players, a clear focus of purpose and some notably high-quality personnel in the different United Nations agencies were identified as the reasons. [27]. Of note is that several ministers have commented that they were relieved that procurement was being managed by the United Nations during this phase because procurement processes have often been used in Zimbabwe to tarnish political opponents.

#### Flexible and pragmatic approaches and evolution of priorities

A potential critique of the New Deal approach outlined in Busan in 2011 is that the goal of an all-inclusive process to establish one vision and one plan is too ambitious in the polarised environments of post-conflict or fragile states. In Zimbabwe, the overall development of the national aid coordination mechanisms has not progressed in more than three years and the national reconciliation commission and constitutional reform processes have also been fraught with problems and delays. Security sector reform did not progress at all prior to the 2013 elections and is now unlikely to be a priority for the new ZANU-PF government. In complex political emergencies, including Afghanistan and Iraq, the multi-donor trust fund model has tended to include a broad range of priorities, from the relatively non-contentious social sectors to the more controversial issues such as justice, constitutional or security sector reform [30]. In such environments, progress in design, launch and implementation tends to occur at the rate of the most controversial governance-oriented programmes. The sector-based transition fund model used in Zimbabwe to date has generally avoided these pitfalls by focusing on realistic and progressive targets in relatively non-controversial areas. The evolution has generally been from a simpler supply-oriented programme (procurement of textbooks or water treatment chemicals or essential medicines) to a more sophisticated scope of work (curriculum reform, rehabilitation of water treatment plants, removal of user fees in the health sector). This evolution of priorities has been tactical as well as strategic. For example, the education sector, had a painful, colonial legacy of discrimination, a highly polarised ministry divided along political lines, and no recent history of large scale donor funding. An initial focus on any programme other than procurement would not have been feasible in the first one to two years. However, phase two of the Educational Transitional Fund has involved training of parent- teacher associations, establishing a new national decentralised mechanism to support direct grants to schools, curriculum reform, and support to robust education management information systems, all designed to improve school governance and accountability at the local level. Such a scope of work would not have been feasible in the initial post-election period. Indeed had a multi-donor trust fund been developed in Zimbabwe in 2008–9, that had included security, judicial and constitutional reform in combination with support to the social sectors, then the entire programme of work would likely have been stalled.

In turn, in each sector, incremental gains have progressively built trust and confidence, as well as contributing to donor harmonisation and alignment, leading to the model now being characterized as a ‘shadow SWAp.’ In addition, because all funding was not locked into one over-arching mechanism, when delays caused by political or operational issues have affected one sector, progress has continued in other sectors. Finally, although the transition funds are designed to progressively build national capacity to manage funding and coordination independently, the mechanism is also flexible enough to continue to operate in much the same way even in the event of a return to a more unstable, humanitarian situation (as was predicted may happen during the election cycle in 2013). This attribute is what has been termed ‘future proofing’ by in-country DFID officials.

#### Emphasis on risk management and risk sharing

The transition funds have provided a relatively ‘safe’ mechanism for donors to fund the social sectors in an otherwise politically and fiscally risky environment. This attribute has in turn helped ensure significant funding for the social sectors at a time when more than 70% of the government budget was allocated for civil service salaries. However, the safety provided requires a very pro-active risk management process. Such a process begins with a strong analysis of potential risks, a shared understanding of the level of tolerance of risk across partners, strong risk communication within and between organisations particularly at ‘crisis’ moments, robust risk mitigation and a sense of solidarity and trust among partners. The latter is particularly important as crises will inevitably occur in such polarised environments; it is critical at these moments that no partner feels that they are bearing all of the risk [31]. Risks that may have not been feasible to take alone may become more manageable if many partners are willing to support a programme. In the context of the transition funds, the pooling of funds has assisted in developing a sense that risks are also ‘pooled’ or shared. A robust response by line ministries, rapid risk communication measures and strong solidarity among partners in Zimbabwe has helped ensure confidence in the integrity of the programmes.

In particular, the capacity for public relations in fragile state environments is often neglected [32]. In the volatile context of Zimbabwe, for example, the highly polarised media has attempted to embroil the transition funds in inter-party political battles on several occasions by making false accusations and sensationalist claims in regards to the programmes supported. However, no serious political interference with the work of the transition funds has been encountered [33] and the progress has continued through the national elections and post-election period. Finally, one practical consideration in managing risk in such environments must be taken into account: managing risks costs money and the riskier the environment, the more it will cost. For example, in the design phase of the new cash grants system of the CPF, the potential risk of local politicians putting pressure on civil servants to favour one geographical area or beneficiary in targeting was assessed as highly significant. In order to mitigate this risk several additional measures were put in place when the programme design was finalised; these measures included contracting out separate and independent providers for each step of the process: the targeting of households; the distribution of the cash; the verification exercise; and the monitoring and evaluation of the programme. While more costly, these mechanisms have ensured the integrity of the programme thus far, and have generated a high degree of confidence in the transparency and fairness of the programme.

#### Emphasis on monitoring and evaluation and sustainability

Given the very high stakes and the high degree of visibility of these programmes in major donor capitals, as well as strong and differing opinions of various constituencies, including the Zimbabwean diaspora and the polarised media, additional investments in internal monitoring and independent evaluation have been required. In order to respond to these needs, UNICEF, in collaboration with the University of Zimbabwe, − with the support of a private philanthropic foundation based in the US (the Nduna Foundation) – has set up a centre (the Collaborative Centre on Operational Research and Evaluation) that can respond to the additional data requirements. In addition to helping rebuild sectoral information systems, the Collaborative Centre supports regular cross-sectional surveys and qualitative research.

Fully fledged capacity building approaches and concrete plans for gradual transfer of management responsibilities have thus far been stymied by tight donor restrictions on funding flowing to government. However, in July 2012, the European Union announced the plan to fully re-activate bilateral aid mechanisms with Zimbabwe by 2014**.** The European Union also announced that the targeted sanctions would be reviewed once certain commitments are met. Other OECD donors are likely to follow suit. Furthermore, prior to the 2013 elections, the World Bank and International Monetary Fund re-engagement process with the Zimbabwe government had begun, possibly leading to the full re-engagement by the Bretton Woods institutions by early 2014.

If donor restrictions dissipate in the future and comprehensive capacity building plans are able to be designed and implemented, it will be critical to monitor, not only the results being achieved through service delivery, but also the policy reforms being successfully negotiated. In addition, monitoring systems should include indicators that capture the improvements in national capacity in technical or programmatic areas as well as in operations areas, such as procurement, human resources planning and public financial management. Furthermore, these monitoring systems will need to capture objectively the effects on donor behaviour in accordance with the commitments made in the Paris Principles and New Deal. Ultimately the aim of all the transition funds is to hand over full responsibility for management to line ministries by the end of 2015. The full implications of the results of the elections in 2013 on these plans remain to be seen.

### Challenges and limitations

The major challenges in implementation of the transition fund model have been the following: a) since discussions are sectorally based and managed, there is a risk that such programmes are not fully captured by Ministry of Finance as ‘on-budget’ or simply result in substitution of domestic resources for aid; b) given the ongoing restrictions imposed by the sanctions environment, there has been a constant tension between donor demands for separate financial management outside government systems and the capacity building requirements of government following years of underinvestment; c) given the results being achieved, there has been a constant need for vigilance in ensuring success is not ‘captured’ by one political party for potential electoral gain resulting in further polarization and backlash.

While Zimbabwe, with its history of effective government social sectors, a well-educated civil service, a highly literate general population, and a sanctions regime in place, certainly represents a specific context, we believe that many of the lessons learned in implementing these funds are potentially generalizable and at least aspects may be replicable to other settings. First several countries, currently facing complex emergencies, such as Syria, Mali, Cote D’ Ivoire, Egypt, Burma, Sudan, and Iraq have a similar recent history of periods of relative stability and functional government systems being in place prior to their current crises. Second, in some cases, sanctions or other donor restrictions have also been imposed. In addition, the model may be useful as an interim financing mechanism to ensure continuity of critical social sector service delivery (without establishing entirely parallel structures) when government-donor relations (temporarily) deteriorate due to political, human rights or accountability concerns. Recent examples of potential applicability in this category include Madagascar, Malawi and Uganda. Finally, in keeping with the current global emphasis on building resilience, we believe that variants of this model could be developed for complex emergencies where shorter cycle humanitarian mechanisms have failed to deliver on longer-term system strengthening and local capacity building objectives (such as Somalia, Chad, South Sudan, etc.). While sustainability is difficult to assess in such chronic emergencies, it is clear that this model may represent a major advance on the primary alternative of using short-term humanitarian funding, mainly through civil society and often in parallel to national institutions and systems. In addition, initial evidence suggests that donor support will remain strong through the period through 2014–15 and that government ownership remains high even if due to the economic situation, it has been difficult for government to increase its domestic financing more significantly. Finally, the authors are not suggesting that this model is applicable for acute humanitarian crises or that it replaces, disease specific mechanisms. We believe this model may be more broadly applicable in chronic emergencies, transitional situations, and for fragile states when more predictability and flexibility of funding is required and where the emphasis should shift towards national capacity building and use of national systems.

## Summary

The New Deal for Engagement in Fragile States agreed at Busan suggests that current ways of working in fragile states need to be revisited. Transitioning out of fragility is a long and challenging process. Processes of political dialogue have often failed due to lack of trust, inclusiveness, and leadership. International partners can often bypass national interests and actors, providing aid in overly technocratic ways that under-estimate the importance of harmonising with the national and local context, and support short-term results at the expense of medium- to long-term goals including capacity building.

The transition fund model has provided a mechanism for development partners to support the social sectors and people of Zimbabwe during a period of complex political transition when donors were not willing to support the Government of Zimbabwe directly. Since 2008, these mechanisms have resulted in significant national scale impact in the major social sectors. In addition to the direct and tangible benefits to the Zimbabwean people, other more indirect impacts on aid alignment and harmonisation as well as policy reform are already becoming evident. The transition fund model is proving to be a useful tool for the current context in Zimbabwe; as of mid-2012, more than US$800 million has been firmly committed to these and related initiatives through 2015. In terms of non-food development assistance, the transition funds now account for around half of all official development assistance to Zimbabwe. As the political situation and the relationship with donors improve in Zimbabwe, the transition funds have the potential to provide a bridge to more traditional development programming. In addition, by focusing on realistic and tangible goals in each of the social sectors, the mechanisms have generally avoided the highly contested issues surrounding control of external funding and been able to demonstrate much more progress, more quickly than other components of the Medium-Term Plan or the overall aid coordination mechanism.

A body of evidence through annual reviews and independent evaluations is accumulating that supports these findings. Formal independent evaluations are planned to document the national impact at the mid-term and end cycle for each fund. This sectoral evidence, combined with an evaluation of the overall effect of the funds on donor harmonisation, alignment and government ownership, will help to provide a formal evidence-base for the use of this model in other countries facing such transitions. The new post-election political reality in Zimbabwe is likely to further test the assumptions and durability of the mechanisms. We would recommend an over-arching, multi-sectoral and fully independent evaluation. Such an evaluation should include a formal review of the role and performance of UNICEF, assess its role in terms of managing potential conflict of interests, efficiency and value for money as well as assessing the potential for other partners to play similar roles in the next phase of the transition funds and in other contexts. If such an evaluation confirms the preliminary findings and final impact assessments, such flexible and pragmatic partnership models have the potential to be institutionalised as a practical method of implementing the New Deal for engagement with fragile states.

## Endnotes

^a^In some cases, donors were not able to formally pool their funds but were able to support the broad programme while retaining some level of earmarking against some of the agreed major result areas.

^b^Humanitarian Plus was a termed used by donors in Zimbabwe from 2009 to indicate their desire to support the social sectors beyond support to purely humanitarian interventions while not straying too close to development which would require a more formal political engagement.
